# Biofuel Production Based on Carbohydrates from Both Brown and Red Macroalgae: Recent Developments in Key Biotechnologies

**DOI:** 10.3390/ijms17020145

**Published:** 2016-02-06

**Authors:** Shigeyuki Kawai, Kousaku Murata

**Affiliations:** 1 Laboratory of Basic and Applied Molecular Biotechnology, Division of Food Science and Biotechnology, Graduate School of Agriculture, Kyoto University, Kyoto 611-0011, Japan; 2 Faculty of Science and Engineering, Department of Life Science, Setsunan University, Osaka 572-8508, Japan

**Keywords:** macroalgae, ethanol, alginate, mannitol, agarose, 3,6-anhydro-l-galactose, *Sphingomonas* sp. A1, *Escherichia coli*, *Saccharomyces cerevisiae*, *Vibrio* sp.

## Abstract

Marine macroalgae (green, red and brown macroalgae) have attracted attention as an alternative source of renewable biomass for producing both fuels and chemicals due to their high content of suitable carbohydrates and to their advantages over terrestrial biomass. However, except for green macroalgae, which contain relatively easily-fermentable glucans as their major carbohydrates, practical utilization of red and brown macroalgae has been regarded as difficult due to the major carbohydrates (alginate and mannitol of brown macroalgae and 3,6-anhydro-l-galactose of red macroalgae) not being easily fermentable. Recently, several key biotechnologies using microbes have been developed enabling utilization of these brown and red macroalgal carbohydrates as carbon sources for the production of fuels (ethanol). In this review, we focus on these recent developments with emphasis on microbiological biotechnologies.

## 1. Introduction

Based on the medium variant projection, the world population of 7.2 billion in mid-2013 is projected to reach 8.1 billion in 2025 and 9.6 billion in 2050 [1]. Thus, the need for research in alternative renewable energy sources is growing each year. Among renewable energy sources, biomass is the only energy source capable of producing liquid fuels [2]. Macroalgae have attracted attention as an alternative source of biomass for the production of both fuels and chemicals. The advantages of macroalgae compared to that of terrestrial biomass includes no requirements for arable land, freshwater, agricultural fertilizer and pesticides. In addition, algae biomass is also relatively easy to extract carbohydrate from, due to the presence of little or no lignin, and has high productivity, and there is little concern for competition with agricultural food and feed crops [2,3,4].

Macroalgae consist of green, red and brown macroalgae. Green macroalgae contain glucans (a polymer of glucose, *i.e.*, cellulose and starch) and sulfated polysaccharides (e.g., ulvan). Red macroalgae contain agar (agarose and agaropectin), carrageenan and glucans. Brown macroalgae contain mannitol, alginate and glucans (cellulose and laminarin) [3]. The contents of glucan based on a dry weight basis in green, red and brown macroalgae are 22% (*Ulva pertusa*), 21.8% (*Gelidium elegans*) and 24.5% (*Alaria crassifolia*), respectively [5]. The macroalgae content of glucan is lower than that in wood (aspen), 45.6% [6], wheat straw, 31.5% [7], and corn stover, 39.5% [8]. However, the content of the other carbohydrates in red and brown macroalgae is higher as described below.

The world production (33 countries) of captured and farmed algae in 2012 is 1.1 million and 23.8 million wet tonnes (tonne = a metric ton, 1000 kilograms), respectively. A few Asian countries dominate the farmed algae production accounting for 96.27% of the total (Table 1) [9].

**Table 1:** Percentage of global farmed algae production.

Country	%
China	53.97
Indonesia	27.40
Philippines	7.36
Korea	4.30
Japan	1.85
Malaysia	1.39
Total	96.27

Farmed algae can be categorized into seven groups (Table 2) [9]. These data show that there has been a rapid increase in the dominance of *Eucheuma* red algae farmed in both tropical and subtropical seawater that is used for carrageenan extraction [9]. Collectively, the major portion of farmed macroalgae is brown and red macroalgae. Since these macroalgae are farmed in Asian countries, a possibility remains that much more macroalgae can be farmed in other countries, including non-Asian countries. Improvement in the farming technology may increase the productivity of macroalgae. Thus, macroalgae, in particular red and brown macroalgae, are undoubtedly a promising renewable energy source capable of producing both liquid fuels and chemicals.

**Table 2:** Categorization of the farmed algae.

The Farmed Algae	Million Wet Tonnes
*Eucheuma* red algae	8.30
Japanese kelp (brown algae, *Laminaria japonica*)	5.65
Seaweed species not identified	2.75
*Gracilaria* spp. (red algae)	2.75
Wakame (brown algae, *Undaria pinnatifida*)	2.10
*Porphyra* spp. (red algae)	1.75
Other seaweeds and microalgae	1.75
Total	25.50

Attempts to produce a high concentration of ethanol (one of the biofuels) from macroalgae have been reviewed [3]. In that review, it was concluded that a conversion of macroalgal glucan into ethanol is apparently not enough, and a conversion of the other carbohydrates (mannitol, alginate, agarose, agaropectin, carrageenan, *etc.*) derived from both brown and red macroalgae into ethanol is needed to achieve a high concentration of ethanol [3]. Although it had been regarded as difficult to convert these brown and red algal carbohydrates into ethanol, recent advances in biotechnology have made it not difficult, as also reviewed recently [10]. However, several developments have been achieved after those reviews were published, and thus, in this review, we further overview this field including the latest developments with emphasis on microbiological biotechnologies.

## 2. Ethanol Production from Carbohydrates in Brown Macroalgae

### 2.1. Carbohydrates in Brown Macroalgae

Brown macroalgae contain alginate, mannitol and laminarin as their major carbohydrates. Laminarin (also called laminaran) is a linear β-1,3-linked glucan (average degree of polymerization from about 15–60) that also contains small amounts of β-1,6-interchain linkages, and the higher degree of this branching makes laminarin more soluble [11]. There are two types of terminal units: one with mannitol (M-series with a non-reducing 1-linked d-mannitol residue) and the other terminated by a reducing glucosyl unit (G-series) present in about a 3:1 ratio [12,13]. The carbohydrate content in brown macroalgae shows seasonal variations, and generally, laminarin is absent during the period of fast growth in the spring, but in both the autumn and winter, it may represent up to 35% of the dried weight of the fronds [13]. Laminarin contents in brown macroalgae are summarized in Table 3. In both the fronds and whole plant, laminarin roughly reached a maximum around September and was at the lowest level in April–June for *L. cloustoni* and in January–April for *L. digitata*, *L. saccharina* in the Northern Hemisphere [14]. β-1,3-glucanases are relatively widespread, and many organisms are able to convert laminarin to glucose, which is a good substrate for fermentation [15]; thus, we do not intensively summarize laminarin in this review.

**Table 3:** Carbohydrates in brown macroalgae [14].

Brown Macroalgae ^a^	Carbohydrates	Parts	Concentration (%)
*L. cloustoni*	Laminarin	Fronds	1–32
		Whole plant	0–18
	Alginate	Fronds	8–18
		Stipes	19–23.5
	Mannitol	Fronds	8–26
		Stipes	5–10
*L. digitata*	Laminarin	Fronds	1–15
		Whole plant	1–12
	Alginate	Fronds	17–25.5
		Stipes	28–33.5
	Mannitol	Fronds	5–23
		Stipes	5–12
*L. saccharina*	Laminarin	Fronds	1–21
		Whole plant	1–18
	Alginate	Fronds	12.5–20
		Stipes	19.5–25
	Mannitol	Fronds	9–23
		Stipes	6–12

**^a^** Brown macroalgae belonging to *Laminaria*.

**Figure 1:**
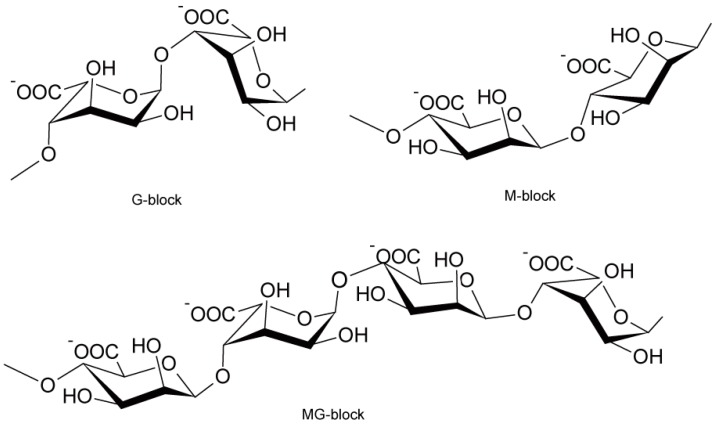
Structure of alginate [3]. G-block: guluronic acid residues; M-block: mannuronic acid residues; MG-block: alternating mannuronic acid and guluronic acid residues.

In contrast to laminarin, key biotechnologies are needed to utilize alginate and mannitol as fermentation substrates. Alginate consists of three types of blocks: the M-block (mannuronic acid residues), the G-block (guluronic acid residues) and the MG-block (alternating mannuronic acid and guluronic acid residues) (Figure 1) [16]. Alginate contents in brown macroalgae are summarized in Table 3. In both fronds and stipes, alginate roughly reaches a maximum in January–March and has the lowest level around September in the Northern Hemisphere [14]. In the case of *Sargassum horneri*, alginate was 26.7%–34.1%, and an obvious seasonal variation was not observed [17]. Although alginate has been regarded as difficult to utilize as a substrate for microbial ethanol production, several developments in new key biotechnologies have been reported as described below.

Mannitol, the sugar alcohol corresponding to mannose, is oxidized to fructose by mannitol-2-dehydrogenase, generating NADH [15]. Some bacteria, such as *Escherichia coli* and *Zymobacter palmae*, can assimilate mannitol [15,18], whereas *Saccharomyces cerevisiae* strains, including the S288C reference strain, are unable to assimilate mannitol for growth, as described below, despite the existence of genes encoding putative homologs of mannitol-2-dehydrogenase (*YEL070W*, *YNR073C*). A few exceptions exist, such as the polyploid strain BB1 [19]. However, two key biotechnologies enabling *S. cerevisiae* to assimilate mannitol have been reported recently [20,21]. Mannitol contents in brown macroalgae are summarized in Table 3. In both fronds and stipes, mannitol roughly reaches a maximum in June–October and has the lowest level in January–April in the Northern Hemisphere [14].

### 2.2. Key Biotechnologies for Production of Ethanol from Mannitol

#### 2.2.1. Ethanol Production from Mannitol Using Microorganisms Other than *S. cerevisiae*

Pioneering work using a bacterium, *Z. palmae* [15], and yeast, *Pichia angophorae* [22,23], developed a method to produce ethanol from mannitol. *Z. palmae* is unable to grow anaerobically in mannitol medium, but under oxygen-limiting conditions, this bacterium produced approximately 12 g·L^−1^ of ethanol in a synthetic mannitol medium containing 38 g·L^−1^ of mannitol with a yield of 0.38 g of ethanol (g mannitol)^−1^ [15]. *P. angophorae* was capable of producing ethanol from 40 g·L^−1^ of xylitol, d-mannitol, d-sorbitol and d-arabitol with a yield of 10.9, 14.4, 9.8 and 1.6 g·L^−1^ of ethanol, respectively [22]. *P. angophorae* has also been shown to assimilate laminarin [23]. *E. coli* is able to assimilate mannitol and to produce ethanol from mannitol. Kim *et al.* demonstrated that *E. coli* KO11 (ATCC24858) produced as much as 25.8 g·L^−1^ of ethanol when provided with 75 and 90 g·L^−1^ of mannitol, resulting in a yield of 0.41 g ethanol (g·mannitol)^−1^ [24].

Forty-five yeast strains other than *P. angophorae* were investigated for the production of ethanol from mannitol, and six (*Saccharomyces paradoxus* NBRC 0259, *Kuraishia capsulata* NBRC 0721, *Kuraishia capsulata* NBRC 0974, *Ogataea glucozyma* NBRC 1472, *Ogataea minuta* NBRC 1473 and *Debaryomyces hansenii* NBRC 0794) of these strains were found to produce ethanol [25]. Of the six strains, *S. paradoxus* NBRC 0259 was selected as the most suitable strain. *S. paradoxus* NBRC 0259 exhibited Ca^2+^-dependent flocculation, especially in the presence of glucose [25]. Yeast flocculation is a reversible, non-sexual cell aggregation in which cells adhere to each other in a Ca^2+^-dependent manner to form flocs. Such flocculation has been used in the brewing industry as a simple and cost-effective way to separate yeast cells from fermentation products [26]. The NBRC 0259 strain required oxygen and intact mitochondrial function to grow in a synthetic mannitol medium, and its ability to produce ethanol from mannitol was enhanced after three days of cultivation in yeast extract/peptone/mannitol liquid medium. The enhanced strain was renamed NBRC 0259-3 [25]. However, such enhancement was not observed in *P. angophorae* and in *E. coli* KO11. Among three strains (NBRC 0259-3, *P. angophorae* and *E. coli* KO11), NBRC 0259-3 exhibited a maximum tolerance to 50 g·L^−1^ ethanol and produced higher amounts of ethanol (40 g·L^−1^) from mannitol than *P. angophorae* did (20 g·L^−1^) in the presence of a high concentration of mannitol (100 g·L^−1^). In the presence of both 20 g·L^−1^ of glucose and 20 g·L^−1^ of mannitol (total sugars, 40 g·L^−1^), all three organisms utilized both glucose and mannitol to produce ethanol, although glucose was more effectively consumed [25].

#### 2.2.2. Ethanol Production from Mannitol Using *S. cerevisiae*

With regard to *S. cerevisiae*, some polyploid strains, such as the BB1 strain, grew in mannitol medium [19]. Growth of the BB1 strain in mannitol medium was dependent on mitochondrial function and required aerobic conditions [19], similar to *S. paradoxus* NBRC 0259 [25]. However, *S. cerevisiae* NCYC231, S288C, BY4742, BY4741, NBRC1346, IAM4512, *Sc41 YJO*, AH109, DBY877, EBY100, SEY6219, T8-1D, YPH500 and SEY6210/6211 strains were unable to assimilate mannitol [19,20,21,25,27,28]. Thus, *S. cerevisiae* had generally been regarded as unable to assimilate mannitol, although *S. cerevisiae* carries two genes encoding putative homologs of mannitol-2-dehydrogenase (*YEL070W*, *YNR073C*) on its chromosome. The molecular basis underlying this inability to assimilate mannitol has remained unknown.

Chujo *et al.* found that cells that had acquired the ability to assimilate mannitol arose spontaneously from wild-type *S. cerevisiae* BY4742 during long culture in either mannitol-containing liquid or solid medium [21]. Chujo *et al.* observed that AH109, DBY877, EBY100, SEY6219, T8-1D and YPH500 strains also acquired this ability in either mannitol-containing liquid or solid medium. The cells that had acquired the ability to assimilate mannitol were tentatively called Mtl+ cells. Most of the Mtl+ cells showed a flocculation phenotype especially in the presence of glucose, and this phenotype was similar to that of *S. paradoxus* NBRC 0259 [25]. The acquisition of mannitol-assimilating ability was attributed to spontaneous mutations in the genes coding for either Tup1 or Cyc8, which constitute a general co-repressor complex that regulates many kinds of genes [21,29]. In other words, those observations strongly suggest that the inability of wild-type *S. cerevisiae* to assimilate mannitol can be attributed to the repressive functions of the Tup1–Cyc8 co-repressor. Some of the Mtl+ cells, such as MK4416, showed no flocculation, even in the presence of glucose. The MK4416 strain carrying the *cyc8Δ1139–1164* allele exhibited higher NaCl-tolerance than that of *P. angophorae* and *E. coli* KO11 and compatible tolerance to that of *S. paradoxus* NBRC 0259-3. The MK4416 strain produced 40 g·L^−1^ of ethanol in the yeast extract/peptone medium containing 100 g·L^−1^ of mannitol [21]. Thus, conferring the ability to assimilate mannitol on *S. cerevisiae* through the dysfunction of Tup1–Cyc8 was succeeded [21].

Enquist-Newman *et al.* obtained three *S. cerevisiae* strains (Lalvin*, Pasteur Red* and SEY/Dip*) that had been induced to grow on mannitol, and identified with a microarray the top three genes induced in the mannitol-containing medium [20]. The three genes encoded mannitol-2-dehydrogenase (Dsf1/YNR073C), putative major facilitator superfamily transporter (Hxt13/Hxt17) and aldose-1-epimerase homolog (YNR071c), and the overexpression of *YNR073C* and *HXT17* was enough to confer the ability on *S. cerevisiae* to produce ethanol from mannitol [20]. Enquist–Newman *et al.* further succeeded in creating the bioengineered *S. cerevisiae* that is able to produce ethanol from a monomeric sugar of alginate, 4-deoxy-l-erythro-5-hexoseulose uronate (DEH), as described below.

### 2.3. Key Biotechnology to Produce Ethanol from Alginate

#### 2.3.1. Alginate Metabolism

**Figure 2:**
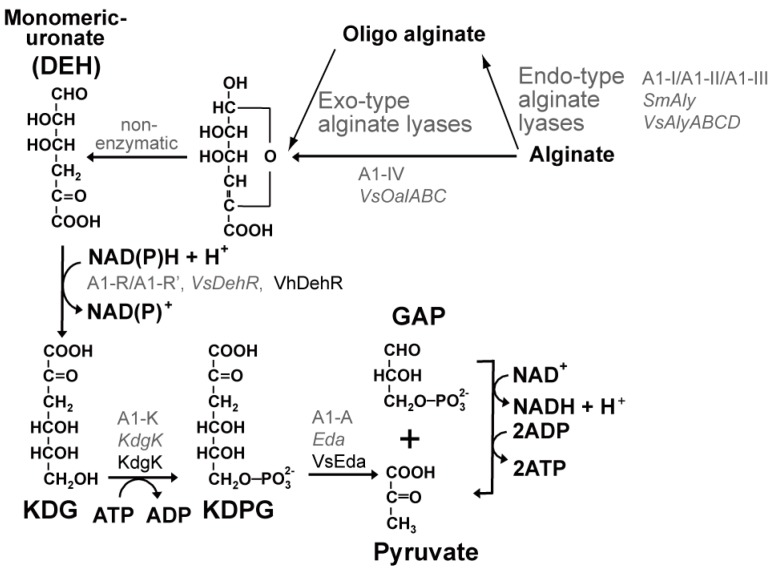
Alginate metabolism. Enzymes present in *Sphingomonas* sp. A1 [31,36,37] are in roman gray; those present or introduced in bioengineered *E. coli* [18] are shown in gray italics; and those introduced in bioengineered *S. cerevisiae* [20] are shown in black. The bioengineered *S. cerevisiae* assimilates 4-deoxy-l-erythro-5-hexoseulose uronate (DEH), but not alginate [20]. Details are described in the text. KDG: 2-keto-3-deoxy-d-gluconate; KDPG: 2-keto-3-deoxy-phosphogluconate; GAP: glyceraldehyde-3-phosphate; VhDehR: *V. harveyi* DehR; VsOalABC: *V. splendidus* 12B01 OalABC; SmAly: *Pseudoalteromonas* sp. SM0524 Aly; VsSlyABCD: *V. splendidus* 12B01 SlyABCD; VsEda: *V. splendidus* 12B01 Eda.

In general, there are a limited number of microbes capable of utilizing alginate. A bacterium, *Sphingomonas* sp. A1, is a remarkable exception that rapidly assimilates alginate [30]. However, this bacterium is unable to assimilate mannitol and was unable to produce ethanol from alginate. The pathway for alginate-metabolism is depicted in Figure 2. Intensive efforts to elucidate the alginate metabolism in this bacterium have greatly contributed to an in depth understanding of this pathway.

In *Sphingomonas* sp. A1, alginate is depolymerized by endo-type alginate lyases (A1-I, A1-II and A1-III) to produce oligo-alginates, which are then degraded by exo-type alginate lyase (A1-IV) into an unsaturated uronate that is further non-enzymatically converted to DEH. Alginate is also directly monomerized to DEH via the unsaturated uronate by the exo-type alginate lyase (A1-IV). DEH is reduced by DEH reductase (A1-R and A1-R’) to 2-keto-3-deoxy-d-gluconate (KDG), which is then phosphorylated by KDG kinase (A1-K) to 2-keto-3-deoxy-phosphogluconate (KDPG). KDPG is cleaved by KDPG aldolase (A1-A) to pyruvate and glyceraldehyde-3-phosphate, which is further metabolized to pyruvate [30,31,32,33] (Figure 2). Among the enzymes involved, genes for KDG kinase (KdgK) and for KDPG aldolase (Eda) are also found in *E. coli*, which is unable to assimilate alginate. These genes are designated as *kdgK* and *eda*, and the latter gene is part of the Entner–Doudoroff pathway [34,35]. DEH reductase is a characteristic enzyme found in alginate-assimilating organisms. The crystal structures of DEH reductases (A1-R and A1-R’) from *Sphingomonas* sp. A1 have been determined [31,36]. A1-R prefers NADPH to NADH, while A1-R’ prefers NADH to NADPH [36], but A1-R’ seems to physiologically utilize both NADH and NADPH (Table 4). The structural requirements responsible for A1-R and A1-R’ to discriminate NADPH from NADH have been carefully analyzed [36].

**Table 4:** *K*_m_ and *K*_cat_ or *V*_max_ of 4-deoxy-l-erythro-5-hexoseulose uronate (DEH) reductases.

Protein	Source Organism	NADPH		NADH		References
		*K*_m_ (mM)	*K*_cat_^a^ or *V*_max_^b^	*K*_m_ (mM)	*K*_cat_^a^ or *V*_max_^b^	
A1-R ^a^	*Sphingomonas* sp. A1	0.009	220	0.192	25	[36]
A1-R’ ^a^	*Sphingomonas* sp. A1	0.272	233	0.024	274	[36]
VsDehR ^b,c^	*V. splendidus* 12B01	2.83	0.08	0.22	0.12	[20]
AtDehR ^b,d^	*A. tumefaciens* C58	0.08	0.05	8.60	0.12	[20]
VhDehR ^b,c^	*Vibrio harveyi*	0.75	0.13	0.37	0.13	[20]

^a^ Steady-state kinetics studies with purified recombinant A1-R and A1-R’ were carried out, and *K*_cat_ (s^−1^) was determined; ^b^ steady-state kinetic studies for these four reductases expressed in *S. cerevisiae* were conducted, and the *V*_max_ (μmol·min^−1^·mg^−1^) was determined; ^c^ the genes for these proteins were codon-optimized for *S. cerevisiae*; ^d^ the gene for AtDehR was not codon-optimized for *S. cerevisiae*.

The exo-type alginate lyase, A1-IV from *Sphingomonas* sp. A1, was the first to be purified and characterized, and the gene for this enzyme was identified [38,39]. The endo-type alginate lyases (A1-I (66 kDa), A1-II (25 kDa), and A1-III (40 kDa)) from *Sphingomonas* sp. A1 seem to be the appropriate enzymes for degrading alginate. A1-I is auto-processed into A1-II and A1-III with different substrate specificities. A1-II degrades polyG and non-acetylated alginate, but does not degrade either polyM or acetylated alginate. A1-III acts on polyM and on acetylated alginate, but not on polyG, whereas A1-III acts on non-acetylated alginate with lesser efficiency (roughly 25%) than that on acetylated alginate. Thus, A1-I shows properties of both A1-II and A1-III [40,41]. These end- and exo-type alginate lyases were purified from intracellular fractions and have been regarded as intracellular enzymes [38,41,42], leading to the critical conclusion that *Sphingomonas* sp. A1 is capable of eating alginate, but not able to eat monouronic acid. The mechanism as to how this bacterium recognizes and eats alginate has been extensively analyzed and reviewed; it has been convincingly determined that the ABC-transporter system is involved in the assimilation of alginate [30,43,44,45], and the quaternary structure of this system has recently been determined [46].

#### 2.3.2. Ethanol Production from Alginate Utilizing Bioengineered *Sphingomonas* sp. A1

To allow *Sphingomonas* sp. A1 to produce ethanol from alginate, Takeda *et al.* introduced two genes encoding pyruvate decarboxylase (Pdc) and alcohol dehydrogenase B (AdhB) from *Zymomonas mobilis*, deleted the gene for lactate dehydrogenase and optimized the condition for the production of ethanol from alginate. Using this approach, Takeda *et al.* succeeded in producing a maximum 1.3 g of ethanol after three days in 100 mL medium containing 5 g sodium alginate with a feeding of 1 g sodium alginate after two days [37]. The medium containing 5% (*w*/*v*) sodium alginate was no longer a liquid medium, but rather a semi-solid medium. However, this bacterium rapidly liquefied this semi-solid medium and produced ethanol, demonstrating the high capacity of this bacterium to assimilate alginate. One of the key biotechnologies for this success was the selection of a strong promoter that was identified using microarray technology [37]. Metabolomics helped to identify the by-pass reaction to be knocked out, leading to the deletion of the lactate dehydrogenase gene and the subsequent improvement in ethanol productivity [37]. Further improvement in ethanol productivity failed due to the accumulation of toxic compounds during fermentation, although a partial alleviation of this toxic effect was overcome by pH adjustment [47]. Break-through technology has been urgently needed to enhance the productivity of ethanol from alginate. Furthermore, it should be noted that this 2011 study is the first report on the production of a valuable compound from alginate.

#### 2.3.3. Ethanol Production from Both Alginate and Mannitol Utilizing Bioengineered *E. coli*

*E. coli* is naturally not able to assimilate alginate, but bioengineered *E. coli* capable of producing ethanol from alginate was successfully constructed [18,48]. Several key technologies were needed to achieve this goal. The initial three key technologies are: (i) an extracellular expression system of endo-type alginate lyase that extracellularly degrades alginate into oligo-alginate; (ii) identification and construction of a gene cluster enabling the transport of oligo-alginate into the cytosol, degradation of oligo-alginate into DEH and conversion of DEH to KDG; and (iii) an introduction of the ethanol pathway to convert pyruvate efficiently into ethanol and to divert carbon flux away from by-products. The extracellular expression system utilized antigen 43 (Ag43), which is an *E. coli* extracellular protein [49], and replaced the native α domain of Ag43 with a truncated alginate lyase plus an aspartyl protease active site. The resulting construct (N455+tSM0524 Aly) was expressed in *E. coli* [18]. The gene cluster consisted of the original gene clusters (*toaA eda kdgK oalBC toaB oalA dehR*) and auxiliary genes (*kdgN toaC alyABC kdgM alyD*) from *Vibrio splendidus* and was integrated into a plasmid (fosmid), yielding pALG3. Expression of pALG3 in *E. coli* permits oligo-alginate to enter the periplasmic space via porin (KdgMN), to be further degraded into oligo-alginate by periplasmic alginate lyases (AlyABCD), to then pass through the cell membrane via a symporter (ToaABC), to be degraded into DEH by exo-type oligo alginate lyase (OalABC) and to be converted to KDG by VsDehR [18]. KDG is then phosphorylated and cleaved to pyruvate and glyceraldehyde-3-phosphate by both kdgK and Eda [18] (Figure 2). The introduction of the ethanol pathway involved integration of *Z. mobilispdc* and *adhB* (genes for Pdc and AdhB) into the chromosome and deletion of *pflB-focA*, *frdABCD* and *ldhA* from the chromosome. Furthermore, the gene clusters (35.3 kb in size) in pALG3 were integrated into the chromosome of several *E. coli* strains, including ATCC8739, DH5α, MG1655, BL21 and W3110, with the help of recombinase-assisted genome engineering (RAGE), and the integrated ATCC8739 exhibiting the best growth in the medium containing degraded alginate was selected as a host. Copies numbers (one or two) of the integrated pALG3 and ethanol pathway (*pdc*, *adhB*) were evaluated, and one copy number of each pALG3 and the ethanol pathway was found to be the best. After integration of N455+tSM0524 Aly into the chromosome, *E. coli* BAL1611 was finally constructed. BAL1611 produced 20 g·L^−1^ of ethanol from 50 g·L^−1^ of a sugar mixture (alginate, mannitol and glucose at a ratio of 5:8:1) and 35–41 g·L^−1^ of ethanol in the 1 L of medium containing 130 g dry milled brown macroalgae (*L. japonica*, kombu) [18,48]. The introduction of the 35.3-kb gene clusters into the chromosome made the clusters stable and was very advantageous for ethanol production resulting in an ~40% improvement of ethanol production over its plasmid-based counterpart at the initial generation and an ~330% margin of ethanol production at generations [48].

#### 2.3.4. Ethanol Production from Both Alginate and Mannitol Utilizing Bioengineered *S. cerevisiae*

The yeast *S. cerevisiae* is the most widely-used microbial cell factory because it has genetic accessibility and robustness under process conditions, and there is considerable fundamental knowledge about the organism [50]. *S. cerevisiae* is unable to assimilate either alginate or DEH, since this microbe does not possess any of the genes required for metabolism of alginate and DEH. Enquist-Newman *et al.* succeeded in getting *S. cerevisiae* to utilize not only DEH, but also mannitol. *YNR073C* and *HXT17* were identified as responsible for mannitol assimilation, as mentioned above, and were chromosomally integrated into *S. cerevisiae* [20]. Three biotechnologies were needed for DEH assimilation: (i) identification of the gene for the DEH transporter (Ac_DHT1); (ii) selection of the best genes for DEH metabolism and integration of all of the required genes, including Ac_DHT1, into the chromosome; and (iii) two adaptations. Ac_DHT1 was identified from the alginolytic eukaryote *Asteromyces cruciatus* through an RNA-sequencing-based differential expression analysis of this fungi grown on alginate *versus* glucose and also through a complementary approach using an *A. cruciatus* cDNA library. For DEH metabolism, genes encoding DEH reductase, KDG kinase and KDPG aldolase are indispensable (Figure 2). The KDG kinase genes from four bacteria (*V. splendidus* 12B01, *E. coli*, *Thermus thermophilus*, *Shewanella frigidimarina* NCIMB 400 and *Saccharophagus degradans* 2–40) and the KDPG aldolase genes from four bacteria (*V. splendidus* 12B01, *Agrobacterium tumefaciens* 0703, *A. tumefaciens* 4944 and *E. coli*) were codon-optimized for and overexpressed in *S. cerevisiae*. The genes of *E. coli* KdgK and *V. splendidus* KDPG aldolase (VsEda) gave the maximum specific activity and were selected and then integrated into the chromosome of *S. cerevisiae* together with the genes for mannitol assimilation. Finally, genes for DEH reductase (VsDehR, AtDehR and VhDehR; Table 4) from three bacteria (*V. splendidus* 12B01, *A. tumefaciens* C58 and *Vibrio harveyi*) were also integrated into the chromosome of the resultant *S. cerevisiae*, yielding BAL2759, BAL2722 and BAL2956. The kinetic parameters of the three reductases expressed in *S. cerevisiae* were also determined (Table 4). Since the three engineered *S. cerevisiae* still showed poor growth in DEH medium, an adaptation experiment was conducted. The initial doubling times (16–64 h) were reduced to 4–5 h after 100–150 generations of subculture over a period of 4–6 months. The microaerobic ethanol production experiments using the adapted strains (BAL2759, BAL2722 and BAL2956) demonstrated that BAL2956 was the best in terms of both ethanol production and consumption of substrates, and thus, BAL2956 was selected. This result also indicated that a DEH reductase capable of using both NADH and NADPH efficiently (VhDehR; Table 4) was crucial. However, since the adapted BAL2956 had a limited capacity for anaerobic ethanol fermentation from DEH and mannitol, a second adaptation experiment was further conducted to get a strain that would grow in both DEH and mannitol under anaerobic conditions. The resultant BAL3215 strain produced 36.2 g·L^−1^ of ethanol from 98 g·L^−1^ of sugar (1:2 molar ratio of DEH:mannitol) [20].

#### 2.3.5. The Key Enzyme for Saccharification of Alginate, an Exo-Type Alginate Lyase

Saccharification of alginate (effective production of DEH from alginate) would also be a key biotechnology when DEH, not alginate, is the substrate, as in the case of the engineered *S. cerevisiae* [20]. The exo-type alginate lyase monomerizes either alginate or oligo-alginate into an unsaturated uronate that is further non-enzymatically converted to DEH. Thus, the exo-type alginate lyase is the key enzyme for saccharification of alginate. Several bacterial exo-type alginate lyases have been well characterized, including A1-IV of *Sphingomonas* sp. A1 [38,39], Atu3025 of *A. tumefaciens* [51,52], Alg17c of *S. degradans* [53,54,55,56] and OalA, OalB and OalC of *V. splendidus* [57]. Genes encoding OalA, OalB and OalC of *V. splendidus* were utilized in bioengineered alginate-assimilating *E. coli* as above [18,57]. A1-IV, Atu3025 and OalA are classified as PL-15, whereas Alg17c, OalB and OalC are classified as PL-17. The tertiary structures of Atu3025 and Alg17c have been determined [54,58], enabling the functions of these enzymes to be improved based on their structural requirements. Recently, it has been reported that Alg7K of *S. degradans*, belonging to PL7 [59], was successfully expressed on the cell surface of *S. cerevisiae* and showed the exo-type alginate lyase activity [60].

## 3. Ethanol Production from Red Macroalgae

### 3.1. Carbohydrates in Red Macroalgae

**Figure 3:**
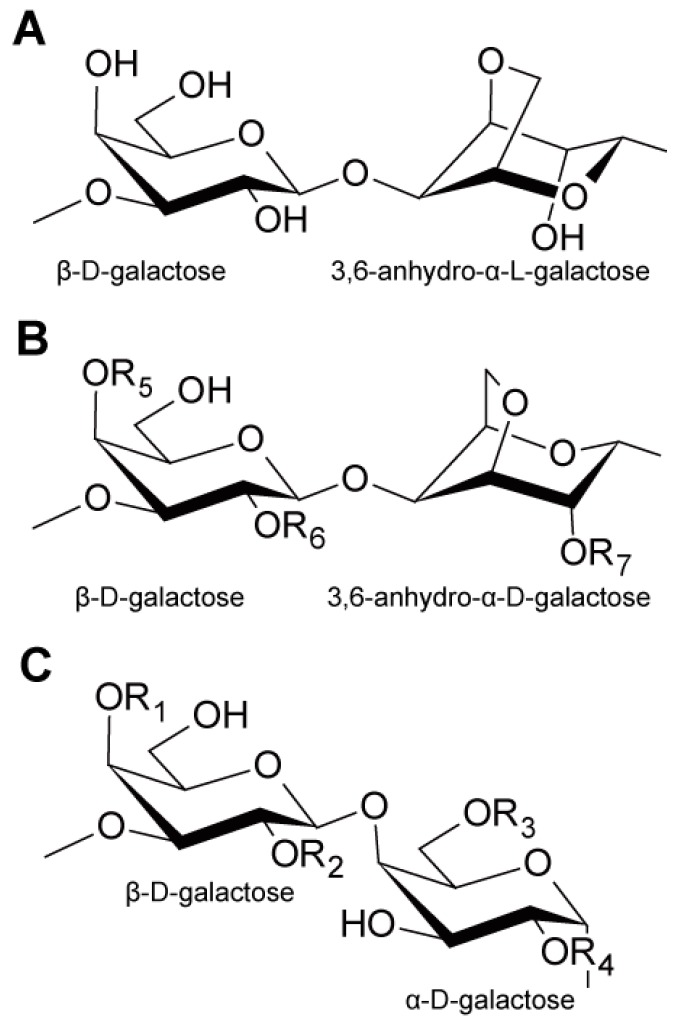
(**A**) Structure of the repeating disaccharide units in agarose; (**B**) structure of the repeating disaccharide units in kappa-carrageenan (R_6_ = R_7_ = H, R_5_ = SO_3_^−^), iota-carrageenan (R_6_ = H, R_5_ = R_7_ = SO_3_^−^) and theta-carrageenan (R_5_ = H, R_6_ = R_7_ = SO_3_^−^); (**C**) structure of the repeating disaccharide units in mu-carrageenan (R_2_ = R_4_ = H, R_1_ = R_3_ = SO_3_^−^), nu-carrageenan (R_2_ = H, R_1_ = R_3_ = R_4_ = SO_3_^−^) and lambda-carrageenan (R_1_ = H, R_2_ = R_3_ = R_4_ = SO_3_^−^) [3].

Red macroalgae contain either agar or carrageenan as their major carbohydrates, in addition to glucans, such as cellulose and floridean starch [3]. Agar consists of agarose and agaropectin [3] and is obtained from red algae, including the commercially important genera *Gelidium* and *Gracilaria* [61]. Agarose is a polysaccharide consisting of repeating disaccharide units composed of β-d-galactose and 3,6-anhydro-α-l-galactose (AHG) (Figure 3A) [3]. Agaropectin has the same repeating units, although some of the l-galactose residues can be replaced with either sulfated galactose residues or partially replaced with 4,6-*o*-(1-carboxyethylidene)-d-galactose [62]. Carrageenans are obtained from different species, such as *Gigartina*, *Chondrus crispus*, *Eucheuma* and *Hypnea* [63]. Carrageenans are traditionally identified by a Greek prefix. Mu-, nu- and lambda-carrageenans mainly consist of repeating disaccharide units composed of β-d-galactose and α-d-galactose, whereas kappa-, iota-, and theta-carrageenans mainly consist of repeating disaccharide units composed of β-d-galactose and 3,6-anhydro-α-d-galactose. These repeating disaccharide units contain some sulfate groups (Figure 3B,C) [3,63]. The three most important commercial carrageenans are iota-, kappa-, and lambda-carrageenans with mu- and nu-carrageenan as the biological precursors of kappa- and iota-carrageenan, respectively [63].

### 3.2. Key Biotechnology to Utilize Agar and Carrageenan for Ethanol Production: Utilization of 3,6-Anhydro-α-l-Galactose (AHG)

A key technology to utilize agar and carrageenan would involve utilization of the common component, AHG. Very recently, Yun *et al.* identified the novel catabolic pathway of AHG in *Vibrio* sp. strain EJY [64]. AHG is oxidized to 3,6-anhydrogalactonate (AHGA) by NADP^+^-dependent AHG dehydrogenase. AHGA is isomerized to 2-keto-3-deoxy-galactonate (KDGal) by AHGA cycloisomerase. When *E. coli* was transformed with the genes coding these novel two enzymes, the *E. coli* transformant acquired the ability to grow in a minimal medium containing AHG as the sole carbon source, although it grew more slowly (reaching an *A*_600_ of 0.4 after 140 h) than it did in glucose medium (reaching an *A*_600_ of 0.5 after 4 h). This study confirmed the activity of the novel pathway *in vivo* [64]. The two genes were also introduced into an ethanologenic *E. coli* KO11, and the resultant KO11 grew in the medium containing 3.2 g·L^−1^ of AHG and 4.1 g·L^−1^ of galactose. The resultant KO11 started to assimilate AHG after initial complete consumption of galactose and finally consumed a 2.0-fold higher concentration of AHG and produced a 1.2-fold higher level of ethanol than that of the control strain carrying the empty vector after 52 h of cultivation [64]. The KDGal was supposed to be phosphorylated and cleaved to pyruvate and glyceraldehyde-3-phosphate and, thus, is expected to participate in the oxidative galactose metabolism pathway, the DeLey–Doudoroff pathway [65,66]. This novel catabolic pathway reminds us of the catabolic pathway of DEH (Figure 2).

### 3.3. Key Biotechnology to Utilize Agar and Carrageenan for Ethanol Production: Improvement in the Utilization of Galactose

**Figure 4:**
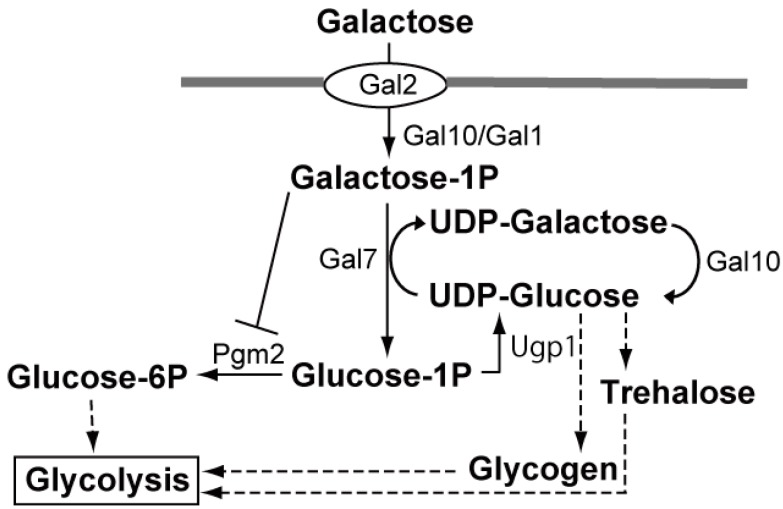
Galactose metabolism in *S. cerevisiae* [67]. Dashed arrows include several reactions. Details are described in the text.

Although galactose is a fermentable sugar, *S. cerevisiae* grows at the half rate in galactose when compared to the growth rate in glucose [67]. Thus, there have been efforts to improve galactose utilization by *S. cerevisiae* [67,68,69,70]. The galactose metabolic pathway is shown in Figure 4 [67]. Galactose is transported into the cell by galactose permease (Gal2) [71]. The conversion of β-d-galactose to glucose-1-phosphate is achieved by the four reactions catalyzed by Gal10, Gal1 and Gal7 that constitute the Leloir pathway [72]. Initially, β-d-galactose is epimerized to α-d-galactose by galactose mutarotase (Gal10) [73]. Galactokinase (Gal1) phosphorylates α-d-galactose to α-d-galactose-1-phosphate [74]. Galactose-1-phosphate uridyl transferase (Gal7) generates glucose-1-phosphate and uridine diphosphate (UDP)-galactose from UDP-d-glucose and α-d-galactose-1-phosphate [75]. UDP-glucose-4-epimerase (Gal10) catalyzes the interconversion of UDP-galactose and UDP-d-glucose to complete the Leloir pathway, where Gal10 is a bifunctional enzyme [76]. Phosphoglucomutase (Pgm2) catalyzes the conversion of α-d-glucose-1-phosphate to α-d-glucose-6-phosphate [77]. Galactose-1-phosphate inhibits Pgm2 [78].

Ostergaard *et al.* either overexpressed *GAL4* or deleted *MIG1*, *GAL80* and *GAL6*, resulting in either the SO7 strain (*GAL4* overexpression) or SO16 strain (∆*gal6* ∆*gal80* ∆*mig1*) [68]. Gal4 is a transcriptional activator, and Mig1, Gal80 and Gal6 play a role in downregulating galactose utilization. The function of Gal4 is inhibited by Gal80, which binds directly to Gal4, and by Mig1, which binds to the promoters of both *GAL4* and *GAL1*, repressing the expression of both genes in the presence of glucose [68,79,80]. Mig1 also recruits the Tup1-Cyc8 complex to the glucose-repressed promoters [29]. Bro *et al.* overexpressed *PGM2*, yielding a PGM2 strain [69]. *PGM2* was identified through microarray analysis in which the transcription of *PGM2* was induced in both SO7 and SO16. No significant changes in the expression of *GAL2*, *GAL1*, *GAL7* and *GAL10* were observed [69]. Hong *et al.* also isolated three mutants (62A, 62B and 62C) through adaptive evolution. The maximum specific growth rate (h^−1^) and ethanol yield (C-mol (C-mol·galactose)^−1^) of the improved strains (SO16, PGM2, 62A, 62B and 62C) were roughly higher than that of the parental strain: 0.17, 0.21, 0.26, 0.26, 0.26 h^−1^ and 0.31, 0.32, 0.14, 0.19 and 0.26 C-mol (C-mol·galactose)^−1^ of the improved strains compared to 0.21 h^−1^ and 0.13 C-mol (C-mol·galactose)^−1^ of the parental strain [67].

Transcriptome analysis of these improved strains showed that transcription of only *PGM2* was induced, while those of other related genes (*GAL2*, *GAL1*, *GAL7*, *GAL10*, *GAL80*, *GAL4*, *GAL3*, *MIG1*, *CYC8*, *TUP1*, *etc.*) were not [67]. The concentrations of glucose-1-phosphate and galactose-1-phosphate in these improved strains were lower than that of the reference strain. The concentrations of glycogen in 62A and in 62C and of trehalose in 62B were higher than that of the reference strain [67]. The authors concluded that Pgm2 plays a key role in controlling the flux through the Leloir pathway, probably due to increased conversion of glucose-1-phosphate to glucose-6-phosphate [67,69]. Overexpression of *PGM2* would partially resolve the problem of feed-forward inhibition of galactose-1-phosphate on Pgm2 [67,78]. Moreover, in the case of the improved strains obtained through an adaptive evolution (62A, 62B and 62C), increasing the flux through either trehalose or glycogen also results in a drain of glucose-1-phosphate and possibly in the increased levels of UDP-glucose, both of which have a positive effect on galactose metabolism [67].

Lee *et al.* investigated the improved strain by introducing a *S. cerevisiae* genomic library on a multi-copy plasmid into *S. cerevisiae* and demonstrated that either overexpression of *SNR84* or truncated *TUP1* is as effective as overexpression of *PGM2* [70]. *SNR84* encodes an H/ACA box small nuclear RNA and is involved in pseudouridylation of ribosomal RNA [70]. Tup1 is a component of a general co-repressor complex that regulates many kinds of genes and seems to be involved in the regulation of galactose metabolism, as mentioned above [29,80]. The truncated Tup1 lacks the *C*-terminal region (281–713 residues) of Tup1 (total of 731 residues) [70].

### 3.4. Key Biotechnology to Utilize Agar and Carrageenan for Ethanol Production: Methods for Saccharification of Agar and Carrageenan

Since the major component of agar is agarose, saccharification methods for agarose have been developed and include: (i) acid hydrolysis; (ii) enzymatic hydrolysis; and (iii) acid pre-hydrolysis followed by enzymatic hydrolysis [81]. The problems with acid hydrolysis and enzymatic hydrolysis are the production of undesired inhibitory compounds, such as 5-hydroxymethylfurfural (5-HMF), and a low yield of monomeric sugar [81,82,83]. The acid pre-hydrolysis method followed by enzymatic hydrolysis seemed the best [83]. In this method, agarose is initially pre-hydrolyzed by non-specific, but preferential cleavages of α-1,3-linkages by a weak acid to even-numbered agaro-oligosaccharides (AOSs) with galactose at the non-reducing ends. AOSs are then enzymatically hydrolyzed to agarotriose, neoagarobiose and the monomeric sugars by a β-agarase II and by neoagarobiose hydrolase (NABH), where the non-reducing end of agarotriose is the galactose unit, whereas that of neoagarobiose is the AHG unit [81]. Agarotriose is the smallest odd-numbered agaro-oligosaccharide [84]. The only problem was that the agarotriose is not further hydrolyzed by β-agarase II and NABH. This problem has been recently solved by identification of a novel agarolytic β-galactosidase that acts on agarotriose and releases galactose [84].

When compared to the case of agarose, saccharification of carrageenan is difficult. Acid hydrolysis produces undesired inhibitory compounds, such as 5-HMF [10]. Enzymatic hydrolysis of carrageenan into monomeric sugars is not possible, since an enzyme that acts on the α-1,3-linkage of neocarrabiose has not been identified. Only endo-type hydrolases capable of cleaving β-1,4-linkages of carrageenan are available [81,85].

## 4. Conclusions and Perspectives

Just a decade ago, it was difficult to convert brown and red algal carbohydrates into ethanol. However, this is now possible due to the development of several key biotechnologies as reviewed here (Table 5). Utilization of agaropectin and carrageenan are not yet possible, and additional research is needed to utilize not only these substrates, but also alginate (or DEH), mannitol and agarose (or AHG). Improvement in the utilization of DEH, mannitol and AHG undoubtedly should be possible as demonstrated by the improvement of the utilization of galactose in *S. cerevisiae*. Expression and secretion of exo-type alginate lyase in bioengineered *S. cerevisiae* is awaited to utilize alginate directly. The method for utilizing glucan, which has been intensively studied for the utilization of terrestrial cellulosic biomass (e.g., [86]), should be integrated into the system described here to comprehensively convert macroalgal carbohydrates into ethanol. Moreover, additional challenges exist for the establishment of a practical system of ethanol production from macroalgae and include overcoming the problems as to where to cultivate macroalgae, how to collect them, how to get carbohydrates from them, how to saccharify some of the carbohydrates (alginate, agar and carrageenan), how to optimize the reaction to produce ethanol, how to scale-up, *etc.* Economical relevance is also a critical matter. Thus, there are many problems to overcome in order to achieve practical utilization of macroalgae. However, taking the advantages of macroalgae over terrestrial biomass into consideration, macroalgae are still a very promising alternative biomass, and further development of the key biotechnologies to utilize macroalgae is expected.

**Table 5:** Summary of the ethanol production in this review.

Strains	Concentration of Sugars in the Medium	Concentration of Ethanol Produced	Reference
*Z. palmae*	38 g·L^−1^ of mannitol	12 g·L^−1^	[15]
*P. angophorae*	40 g·L^−1^ of mannitol	14.4 g·L^−1^	[22]
*E. coli* KO11	75 g·L^−1^ of mannitol	25.8 g·L^−1^	[24]
*S. paradoxus* NBRC 0259-3	100 g·L^−1^ of mannitol	45 g·L^−1^	[25]
*S. cerevisiae* MK4416	100 g·L^−1^ of mannitol	40 g·L^−1^	[21]
Bioengineered *Sphingomonas* sp. A1	(50 g + 10 g)·L^−1^ of sodium alginate	13 g·L^−1^	[37]
Bioengineered *E. coli* BAL1611	50 g·L^−1^ of a sugar mixture (alginate, mannitol, and glucose at a ratio of 5:8:1)	20 g·L^−1^	[18,48]
Bioengineered *E. coli* BAL1611	130 g·L^−1^ of dry milled brown macroalgae (*L. japonica*, kombu)	35–41 g·L^−1^	[18,48]
Bioengineered *S. cerevisiae* BAL3215	98 g·L^−1^ of sugar (1:2 molar ratio of DEH:mannitol)	36.2 g·L^−1^	[20]
Bioengineered *E. coli* KO11	3.2 g·L^−1^ of AHG and 4.1 g·L^−1^ of galactose	1.4 g·L^−1^	[64]
